# Gonococcal endocarditis in a 32-year-old male: a rare presentation of an underrecognized disease – case report

**DOI:** 10.1097/MS9.0000000000001125

**Published:** 2023-09-04

**Authors:** Lila H. Abu-Hilal, Yumna Njoum, Farah M. Jabbarin, Duha I. Barghouthi, Abdullah Hamamdah, Mohammad Bourini, Sameer Mtour

**Affiliations:** a Faculty of Medicine, Al-Quds University, Jerusalem, Palestine; b Cardiology Department; c Internal Medicine Department; d Nephrology Department, Al-Makassed Hospital, Jerusalem, Palestine

**Keywords:** case report, disseminated gonococcal infection, infective endocarditis, *Neisseria gonorrhoeae*

## Abstract

**Introduction and importance:**

Disseminated gonococcal infection (DGI) is an infrequent but serious complication of gonorrhea that can exhibit atypical symptoms. While rare, it can lead to infective endocarditis (IE), a condition that affects the heart valves and can result in severe and potentially life-threatening outcomes.

**Case presentation:**

We present a case of *Neisseria gonorrhoeae*-caused IE confirmed by blood culture and direct isolation from the aortic valve vegetation. Our patient experienced complications, including glomerulonephritis, respiratory failure, and positive troponin. Urgent surgery successfully removed a large vegetation, replaced the aortic valve, and improved cardiac function. Follow-up showed symptom resolution.

**Clinical discussion:**

DGI can present atypically with a triad of tenosynovitis, polyarthralgia, and rash, even without genitourinary symptoms. However, it can also present with nonspecific symptoms, leading to a later diagnosis of IE, as observed in our patient, who developed an aortic valve abscess and aortic regurgitation.

**Conclusion:**

This case provides important insights into the diagnosis and management of gonococcal endocarditis, emphasizing the significance of early recognition, timely intervention, and multidisciplinary collaboration in improving patient outcomes. It is imperative to have a high level of suspicion for this rare entity, given its high virulence and potential for severe complications.

## Introduction

Highlights

- Gonococcal endocarditis in a young male, presenting with constitutional symptoms.
- Successful multidisciplinary management of infective endocarditis.
- Early recognition and timely intervention improve patient outcomes.
- Diagnostic challenges and management strategies for gonococcal endocarditis.
- Consider gonococcal infection in symptomatic patients with recent sexual activity.

Gonorrhea is a sexually transmitted disease (STD) caused by the bacterium *Neisseria gonorrhoeae*. It can infect the mucous membranes of the genitourinary tract, eye, rectum, and throat. This infection is a major public health concern, and it is estimated that there are over 400 000 cases of gonorrhea in the United States each year, but fewer than half are reported^1^.

Disseminated gonococcal infection (DGI) is a rare manifestation of gonorrhea, occurring in only 0.5–3% of cases when the bacteria spread through the bloodstream. DGI may present with atypical symptoms, such as tenosynovitis, polyarthralgia, and rash, in the absence of genitourinary symptoms. However, in 1% of cases, it can cause infective endocarditis (IE) involving heart valves, which can have severe and potentially fatal consequences^2^.

Here, we present the case of a 32-year-old male patient with symptoms of fever, fatigue, and shortness of breath. Despite the absence of genitourinary symptoms, the patient was diagnosed with gonococcal IE following thorough evaluation and testing that was early enough to manage the life-threatening complications he developed.

This case has been reported in line with the SCARE (Surgical CAse REport) criteria^3^.

## Case presentation

A 32-year-old male patient without significant past history presented with a 2-week history of weakness, fatigue, intermittent low-grade fever reaching 38.1°C with chills and rigor, decreased appetite, and exertional shortness of breath. He denied cough, urogenital symptoms, skin rashes, hemoptysis, headache, joint pain, or swelling. He is a smoker, has multiple tattoos, and is sexually active with different partners but has no history of alcohol or recreational drug use.

On examination, he was stable vitally except for a temperature of 38.7°C and a respiratory rate of 21 breaths per minute. He was in pain and diaphoretic. There were no crackles or wheezes on lung examination; a grade 2 early diastolic murmur was heard at the left lower sternal border. No meningeal signs, focal neurological deficits, lower limb edema, or skin lesions were observed.

Initial workup revealed a C-reactive protein of 105 mg/l, erythrocyte sedimentation rate of 8 mm/h by the end of the first hour, and urinalysis showed many white blood cells, few bacteria, and many red blood cells. Creatinine was 7.32 mg/dl with an estimated glomerular filtration rate of 8.7 ml/min/1.73 m^2^ according to the MDRD (modification of diet in renal disease) equation, and chest X-ray was normal.

He received empirical treatment with meropenem and vancomycin for 5 days as a sepsis case, following blood and urine cultures. There were suspicions of systemic lupus erythematosus with Libman–Sacks endocarditis. The patient exhibited low C3 and C4 levels, inconclusive antinuclear antibodies screen (ELISA – enzyme-linked immunosorbent assay), and negative results for double-stranded DNA, anti-Smith, P-ANCA (perinuclear antineutrophil cytoplasmic antibodies), C-ANCA (cytoplasmic antineutrophil cytoplasmic antibodies), anti-GBM (anti-glomerular basement membrane antibodies), and rheumatoid factor. While urine culture was negative, blood culture revealed *Neisseria gonorrhoeae*, leading to a 10-day course of ceftriaxone based on culture and sensitivity tests.

**Figure 1:**
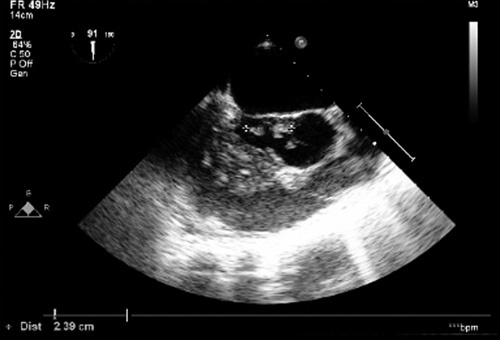
Aortic valve vegetation, seen with transthoracic echocardiogram.

Transthoracic echocardiogram was done to rule out IE as a cause of the patient’s manifestations, and it showed an echogenic mass 1.4×1 cm with severe aortic regurgitation (Fig. 1).

A transesophageal echocardiogram confirmed an aortic valve abscess and a large enough vegetation to cause the aortic regurgitation and cause the clinical picture of acute decompensated heart failure.

**Figure 2:**
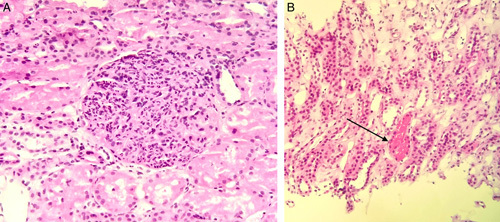
(A) Hematoxylin and eosin (H&E): 20× – diffusely enlarged and hypercellular glomeruli due to neutrophils and macrophages along with the proliferation of mesangial and endothelial cells. (B) H&E: 20× – tubules contain red blood cells (arrow).

A renal ultrasound was done to evaluate the chronicity of the renal impairment, which showed normal kidney size bilaterally and normal corticomedullary differentiation with no hydronephrosis warranting a kidney biopsy, which revealed diffuse endocapillary proliferative glomerulonephritis. Immunofluorescence was performed, which showed negative immunoglobulins IgA, IgM, and complement components C4 with +1 peripheral granular IgG and +2 peripheral granular C3 (Fig. 2).

On the third day during hospitalization, the patient complained of chest pain, and an electrocardiogram revealed variable PR prolongation without depression or ST segment deviation. Troponin levels were positive in two sets 6 h apart, indicating cardiac ischemia. Vital signs revealed hemodynamic compromise, so the patient was transferred to the cardiac care unit for monitoring and was found to have a wide pulse pressure and an average blood pressure of 118/35, indicating aortic valve regurgitation and urgent surgery was planned.

However, before the procedure, the patient experienced increasing shortness of breath, increased oxygen requirements, and decreased urine output. A portable chest X-ray revealed signs of volume overload and pulmonary congestion. Despite non-invasive ventilation, the patient’s condition deteriorated; he was intubated and transferred to the intensive care unit due to severe respiratory distress caused by acute decompensated heart failure. Oxygen saturation reached 86% despite maximum continuous positive airway pressure settings. Acute kidney injury and volume overload necessitated hemodialysis using a femoral gamma catheter, as intravenous diuresis was ineffective.

The patient underwent successful verruca excision and aortic valve replacement after stabilization and obtaining informed consent. Intraoperatively, an aortic valve abscess and vegetation were discovered, and tissue cultures confirmed the presence of *N. gonorrhea* (*Neisseria gonorrhoeae*), confirming the diagnosis of IE. The patient was discharged 5 days post-operation without any immediate complications. During the subsequent 3-month follow-up period, the patient remained completely asymptomatic and reported an excellent improvement in previous symptoms. Physical examination revealed normal vital signs and resolved murmur. Follow-up echocardiography confirmed complete normalized cardiac function. Kidney function is now normal, and 9 months later, the patient remains stable.

## Discussion

*N. gonorrhoeae* is a gram-negative diplococcus and a pathogen that exclusively affects humans. It is responsible for the STD, gonorrhea, which can have serious complications that require careful study^4^.

DGI is a rare complication of *N. gonorrhoeae* infection, usually secondary to local inflammatory tissue damage leading to the protected transmission of *N. gonorrhoeae* inside the neutrophils into the bloodstream^5^. It is characterized by a classic triad of dermatitis, tenosynovitis, and migratory polyarthritis. While DGI is more frequently seen in young women, it can affect sexually active individuals of any age. The initial mucosal infection is often asymptomatic, which can make the diagnosis challenging as there may not be any apparent infectious cause for the symptoms^6^.

DGI can present in various forms, often without preceding genitourinary symptoms^7^, including Gonococcal endocarditis, which is an uncommon manifestation of DGI, with only a few reported cases in the literature. It typically presents subacutely after around 2–4 weeks with fatigue, two fever spikes per day, also known as double quotidian fevers, chills, jaundice when the liver is involved, preceding arthritis, petechial rash, renal dysfunction, and new cardiac murmurs^8,9^.

Early diagnosis and prompt treatment of gonorrhea can prevent the development of DGI and endocarditis. Treatment typically involves antibiotics, and individuals should be tested for other STDs as well. Safe sex practices, such as consistent condom use, can help prevent its transmission^1^.

Our patient presented with a complex set of symptoms that posed a diagnostic challenge. The nonspecific nature of the symptoms made it difficult to narrow down the differential diagnoses. However, after conducting laboratory tests, we discovered severe renal impairment, leading to a diagnosis of acute glomerulonephritis, a condition not typically associated with the presenting symptoms. Additionally, the patient had an aortic valve abscess, adding to the uniqueness and complexity of the case. Our findings underscore the importance of considering a broad range of potential diagnoses and conducting a comprehensive workup in cases of atypical symptoms or laboratory findings.

## Conclusion

This case highlights the importance of considering gonococcal infection in patients presenting with fever and atypical symptoms, particularly in those with a history of recent sexual activity or other risk factors for STDs. It also underscores the potential severity of DGI and the need for prompt diagnosis and treatment. These findings have important implications for clinical practice and may inform the development of more effective diagnostic and treatment strategies for gonococcal infections.

## Ethical approval

This case report was conducted at Al-Makassed Hospital, and it is important to note that the study is exempt from ethical approval in our institution. The exemption was granted based on the nature of the study, which involves a single case report and does not involve experimental interventions or additional procedures beyond routine clinical care. Patient approval was obtained for the publication of this case report, and all necessary measures were taken to ensure patient confidentiality and privacy.

## Consent

Written informed consent was obtained from the patient for the publication of this case report and accompanying images. A copy of the written consent is available for review by the Editor-in-Chief of this journal on request.

## Sources of funding

None.

## Author contribution

L.H.A.-H.: was responsible for the abstract, introduction, discussion, and editing the case presentation; Y.N., F.M.J., and A.H.: were responsible for the case presentation and assisted with the revision process; D.I.B.: assisted with the revision process; S.M. and M.B.: provided critical feedback on the manuscript. All authors participated in concept development, revision of the manuscript, and read and approved the final manuscript.

## Conflicts of interest disclosure

The authors declare no conflicts of interest.

## Research registration unique identifying number (UIN)

1. Name of the registry: Research Registry.
2. Unique identifying number or registration ID: researchregistry9098.

## Guarantor

Dr Sameer Mtour.

## Data availability statement

The original contributions presented in the study are included in the article/supplementary material; further inquiries can be directed to the corresponding authors.

## Provenance and peer review

Not commissioned, externally peer-reviewed.
